# Questioning the “SPIN and SNOUT” rule in clinical testing

**DOI:** 10.1186/s40945-019-0056-5

**Published:** 2019-03-07

**Authors:** Jean-Pierre Baeyens, Ben Serrien, Maggie Goossens, Ron Clijsen

**Affiliations:** 1 Faculty Physical Education and Physiotherapy, Vrije Universiteit Brussel, Pleinlaan 2, 1050 Brussel, Belgium; 2 International University of Applied Sciences THIM, Weststrasse 8, 7302 Landquart, Switzerland; 3 Faculty Applied Engineering, Antwerp University, Campus Groenenborgerlaan 171, G.U. 148, 2020 Antwerpen, Belgium; 4 Department of Business Economics, Health and Social Care, SUPSI, Weststrasse 8, 7302 Landquart, Switzerland

**Keywords:** Clinical testing, Diagnostic accuracy, Sensitivity, Specificity, Likelihood ratio, Prevalence

## Abstract

Specificity (SP) and sensitivity (SE) answer the question ‘what is the chance of a positive or negative test in response to the presence or absence of a clinical condition?’. Related to SP and SE are the diagnostic procedures of SNOUT and SPIN. SNOUT is the acronym for ‘Sensitive test when Negative rules OUT the disease’, SPIN for, ‘Specific test when Positive rules IN the disease’. SE and SP are incomplete because for clinical diagnosis, the question of concern should actually be: ‘what is the chance that the clinical condition will be present or absent in the context of a positive or negative test result?’. The latter statement is related to the concepts of Positive and Negative Predictive Value (PPV and NPV). However, PPV and NPV are predictive values not only dependent on SE and SP but also largely dependent on the prevalence in the examined population. Consequently, predictive values from one study should not be transferred to some other setting with a different prevalence. Prevalence affects PPV and NPV differently. PPV is increasing, while NPV decreases with the increase of the prevalence. This makes prevalence the nemesis in the application of the predictive values. Therefore, another variable has been introduced to evaluate the strength of a diagnostic test, namely the likelihood ratio. Likelihood ratios determine how much more likely a particular test result is among people who have the clinical condition of interest than it is among people who do not have the condition. LIKELIHOOD RATIO (LR) is the ratio of two probabilities. This letter illustrates the limitations of the concepts of SE, SP, NPV, PPV and the LRs in context of specific shoulder tests.

## Background

Diagnosis is a tool/skill/procedure to guide your choice into best therapy [1]. Errors in diagnosis may lead to non-treatment of a clinical entity being present or treatment of lesions which actually are not present.

A clinical test can be positive (+) or negative (−) related to the presence [1] or absence (0) of a clinical condition of interest. The presence of a clinical condition is also called ‘event’ or ‘target event’. Diagnostic index tests are confronted with uncertainty concerning the presence or absence of a clinical condition and have to deal with ‘False positives’ and ‘False negatives’ outcomes. The relation between a positive or negative test and the presence or absence of the clinical condition can be presented in a 2*2 cross table (also called contingency table) Table 1.

An overall definition reflecting the strength of a diagnostic test is the ‘Diagnostic Accuracy’ defined as

$$ \mathrm{DA}=\frac{d1+h0}{n} $$

SENSITIVITY (SE) and SPECIFICITY (SP) describe the diagnostic performance of a test in a group of patients by comparing the result of the test with whether the condition of interest is actually present as indicated by a reference ‘golden’ standard [2].

SE refers to the strength of a clinical test to correctly represent the clinical entity of a patient:

$$ \mathrm{SE}=\frac{True\ positives}{True\ positives+ False\ negatives}=\frac{d1}{n1.} $$

A sensitivity of 0.75 means that 75% of the patients having the clinical entity (True Positives + False Negatives) will be identified with the test (True Positives), but also that 25% remain undetected with the test (False Negatives). SP refers to the potency of a clinical test to correctly represent those patients not having the clinical entity:

$$ \mathrm{SP}=\frac{True\ negatives}{True\ negatives+ False\ positives}=\frac{h0}{n0} $$

A specificity of 0.83 means that 83% of the patients not having the clinical entity (True Negatives +False Positives) will be tested correctly (True Negatives), but also that 17% of them will be wrongly stigmatized as having the clinical condition (False Positives).

Other terms related to sensitivity and specificity used in journals are:

SE = ‘True Positive rate’ = ‘True Positive Fraction’ (TPF)

(1-SE) = ‘False Negative Fraction’ (FNF)

SP = ‘True Negative Fraction’ (TNF)

(1-SP) = ‘False Positive Rate’ = ‘False Positive Fraction’ (FPF)

The (1-α) confidence intervals for SE and SP follow a normal distribution and are respectively

$SE\pm {z}_{\alpha /2}\sqrt{\frac{SE\ast \left(1- SE\right)}{n1}}$ and $SP\pm {z}_{\alpha /2}\sqrt{\frac{SP\ast \left(1- SP\ \right)}{n0}}$, with *z*_*α*/2_ the critical z-scores with α the level of significance.

Overall, SE and SP values of clinical tests for a specific clinical event as reported in research journals range highly due to the heterogeneity of the research samples differing in clinical features such as level or type of the event or comorbidity components. In the literature regarding the shoulder lesions, for example, high diagnostic values of test are due to the fact that the orthopaedic surgeon applies the test when the patients have already been screened and selected so that they constitute a niche in which the test performs very well [3]. A meta-analysis on SLAP testing presented for instance for the O’Brien test 95% confidence intervals for SE and SP of respectively (0.55–0.75) and (0.21–0.55) with pooled results for SE and SP of respectively 0.66 and 0.36 [4]. Spectrum bias presents itself when the spectrum of patients in the research sample is not representative of patients seen in clinical practice, for instance when the research sample is excluded with borderline or mild expressions of the clinical entity. Spectrum bias overestimates the accuracy of the test [5]. With verification bias the results of a diagnostic test affect the use of the golden standard test. With verification bias, the golden standard test is not applied consistently to confirm negative results of the index test. These patients are then excluded from the sample or considered true negatives. Verification bias may overestimate the SE and underestimate the SP or overestimate the SE as well as the SP [6]. An important reason why sensitivity and specificity are incomplete for clinical diagnosis is that they answer the wrong question, i.e. wrong in a clinical practice context. Specificity and sensitivity answer the question ‘what is the chance of a positive or negative test in response to the presence or absence of a clinical condition?’. For clinical practice, the question of concern should actually be: ‘what is the chance that the clinical condition will be present or absent in context of a positive or negative test result?’.

**Table 1:** Contingency table between a clinical condition and clinical index test result

Test result	Cinical condition	1	0	
+		d1	d0	d = n^+^
–		h1	h0	h = n^−^
		n1	n0	n

***‘True positives****’ = d1 = the patient has the clinical condition* [1] *and the clinical test is positive (+)*

***‘False positives’*** *= d0 = the patient does not have the clinical condition (0) but the clinical test is positive (+)*

***‘True negatives’*** *= h0 = the patient does not have the clinical condition (0) and the clinical test is negative (−)*

***‘False negatives’*** *= h1 = the patient has the clinical condition* [1] *and the clinical test is negative (−)*

***d = sum of ‘True Positives’ & ‘False Positives’***

***h = sum of ‘True negatives’ & ‘false negatives’***

Let us put this question in context of probability theory. Given the probability P [1] as the probability of the presence of a clinical condition in a population (i.e. the prevalence = $\frac{n1}{n}$), and P(+) the probability of a clinical index test being positive (i.e. $\frac{d}{n}$). P(+∖1) is then defined as the conditional probability of a positive test (+) as dependent on the presence of the clinical condition [1]. This is the SE of the test. However, from a clinical context the relevant question is: ‘what is the chance that the clinical condition will be present [1] when a positive test (+) result is present, i.e. P(1 ∖+)?’. With ‘∩^′^ the symbol for the Boolean operator ‘and’, probability theory states that $\mathrm{P}\left(1\setminus +\right)=\frac{P\left(1\cap +\right)}{P\left(+\right)}=\frac{d1/n}{d/n}=\frac{d1}{d}$and $\mathrm{P}\left(+\setminus 1\right)=\frac{P\left(1\cap +\right)}{P(1)}=\frac{d1/n}{n1/n}=\frac{d1}{n1}$.

From these equations the theorem of Bayes can be deduced: $\mathrm{P}\left(1\setminus +\right)=\frac{\mathrm{P}\left(+\setminus 1\right)\ast P(1)}{P\left(+\right)} = \frac{\frac{d1}{n1}\ast \frac{n1}{n}}{\frac{d}{n}}=\frac{\frac{d1}{n}\ }{\frac{d}{n}}=\frac{d1}{d}$

The prevalence P [1] is called a priori probability and the conditional statement P(1∖ + ) a posteriori probability.

$\mathrm{P}\left(1\setminus +\right)=\frac{d1}{d}$ is defined as Positive Predictive Value = PPV = ‘POST-test probability for a POSITIVE test’ =

$$ \frac{True\ Positives}{True\ Positives+ False\ Positves}=\frac{d1}{d} $$

Important to accentuate is that the conditional probability P(+∖1) ≠P(1∖ + )! In other words, SE is not the same as PPV. The output of PPV is dependent on the prevalence P [1], the SE and the SP of the test:

$$ \mathrm{PPV}=\mathrm{PREVALENCE}\ast \frac{sensitivity}{PREVALENCE\ast sensitivity+\left(1- PREVALENCE\right)\ast \left(1- specificity\right)}=P(1)\ast \frac{SE}{P(1)\ast SE+\left(1-P(1)\right)\ast \left(1- SP\right)}\cdotp $$

For instance, with a high SE of 0.9, a SP of 0.4 and a prevalence of 0.30, the PPV is 0.4. There is a 40% chance of presence of the clinical condition with a positive test. In contrast to the high sensitivity, this is quite confronting. Another example: The prevalence of SLAP lesions has been reported between 6 and 26% [7]. For the O’Brien test (used to detect a SLAP lesion), taking into account the pooled results for SE and SP of respectively 0.66 and 0.36, PPV would range between 6% (for a prevalence of 6%) and 27% (for a prevalence of 26%) [8].

Table 2 presents a Monte Carlo simulation for PPV related to differences in prevalence (P [1]), SE and SP, categorized in four combinations of SE and SP (0.8_0.8; 0.8_0.4; 0.4_0.8; 0.4_04) and four levels of prevalence (0.2, 0.4, 0.6, 0.8). The table reveals the impact of the prevalence on the PPV values. The table also demonstrates that the PPV is not uniquely related to the SE but on the combination of SE and SP. For instance, the combination of a low SE and high SP (0.4_0.8) presents higher PPV values than the combination of a high SE and low SP (0.8_0.4).

Initiated by Sacket et al. [9] it is commonly believed that if a very high SP is present the SPIN rule can be of use. SPIN is the acronym for ‘Specific test when Positive rules IN the disease’. The rationale behind the SPIN rule is that a test with a high SP is very specific with what it tests for, it is good at excluding the clinical condition. So, if the test has a high SP and the result is positive one can be nearly certain that the clinical condition is present.

The SPIN rule is a statement from the point of view of the conditional statement P(1∖ + )= PPV.

SPIN relates a high SP to an acceptable PPV. However, as can be seen in Table 2, the ability to rule in depends not only on the SP but also on the SE AND the prevalence. Consequently, the SPIN rule is not applicable when the prevalence is low. Furthermore, the PPV is reduced when a high SP is combined with a low SE (for instance PPV = 0.57 under a prevalence of 0.4 for SE_SP 0.4_0.8 as compared to PPV = 0.73 for SE_SP 0.8_0.8 under same prevalence of 0.4).

**Table 2:** PPV values in relation to prevalence, SE and SP values SE_SP = value of SE _ values of SP

PPV		PREVALENCE			
		0.2	0.4	0.6	0.8
SE_SP	0.8_0.8	0.50	0.73	0.86	0.94
	0.4_0.8	0.33	0.57	0.75	0.89
	0.8_0.4	0.25	0.47	0.67	0.84
	0.4_0.4	0.14	0.31	0.50	0.73

The same reasoning can be applied on the statement P(0∖ − ),i.e. ‘What is the chance that the clinical event will not be present (0) when a negative test (-) result is present?’ Given the probability P(0) as the proportion of healthy people for the target disorder (i.e. P(0) = 1 – prevalence = 1 – P [1], = $\frac{n0}{n}$), and P(−) the probability of a clinical index test being negative (i.e. $\frac{h}{n}$). Then $\mathrm{P}\left(0\setminus -\right)=\frac{P\left(0\cap -\right)}{P\left(-\right)}=\frac{h0/n}{h/n}=\frac{h0}{h}=\mathrm{NPV}$ and $\mathrm{P}\left(-\setminus 0\right)=\frac{P\left(0\cap -\right)}{P\left(-\right)}=\frac{h0/n}{n0/n}=\frac{h0}{h0}=\mathrm{SP}$. Here also, P(−∖0) ≠P(0∖ − )! As well as the PPV, the NPV is dependent on the prevalence P [1], the SE and the SP of the test:

$$ \mathrm{NPV}=\frac{\left(1-\mathrm{PREVALENCE}\right)\ast specificity}{PREVALENCE\ast \left(1- sensitivity\right)+\left(1- PREVALENCE\right)\ast specificity}=\frac{\left(1-P(1)\right)\ast SP}{P(1)\ast \left(1- SE\right)+\left(1-P(1)\right)\ast SP} $$

1-NPV is termed the ‘POST- test probability of a NEGATIVE test’.

For example: For the O’Brien test, taking into account the pooled results for SE and SP of respectively 0.66 and 0.36, NPV would range between 94% (for a prevalence of 6%) and 75% (for a prevalence of 26%) [4]. Two peculiar things are presented here: the bigger the prevalence the smaller the NPV and despite a small SP, the NPV is relatively larger. This needs clarification.

Table 3 presents a Monte Carlo simulation for NPV related to differences in prevalence (P [1]), SE and SP, categorized in four combinations of SE and SP (0.8_0.8; 0.8_0.4; 0.4_0.8; 0.4_0.4) and four levels of prevalence (0.2, 0.4, 0.6, 0.8). The table reveals the impact of the prevalence on the NPV values, but opposite to the PPV NPV decreases with an increase in prevalence. The table also demonstrates that the NPV is not uniquely related to the SE but merely on the combination of SE and SP. For instance, the combination of a low SE and high SP (0.4_0.8) presents higher NPV values than the combination of a high SE and low SP (0.8_0.4), which is opposite to the finding for PPV (as mentioned above).

**Table 3:** NPV values in relation to prevalence, SE and SP values SE_SP = value of SE _ values of SP

NPV		PREVALENCE			
		0.2	0.4	0.6	0.8
SE_SP	0.8_0.8	0.94	0.86	0.73	0.50
	0.4_0.8	0.84	0.67	0.47	0.25
	0.8_0.4	0.89	0.75	0.57	0.33
	0.4_0.4	0.73	0.50	0.31	0.14

SNOUT is the acronym for ‘Sensitive test when Negative rules OUT the disease’ [9]. The rationale behind the SNOUT rule is that if a test has a high sensitivity, one can be confident that it will detect the clinical event and so if the test result is negative, one can be nearly certain that the clinical condition is not present. The SNOUT rule is actually a statement from the point of view of the conditional statement P(0∖-) = NPV. SNOUT intrinsically relates a high SE to an acceptable NPV. From the table, it is clear that this is only applicable for the lower prevalences. Furthermore, a low SE can also be associated to an acceptable NPV under the condition of low prevalence. Consequently, the SNOUT rule can be very misguiding within the diagnostic process. Considering the abovementioned arguments, the use of SE and SP should be avoided in clinical diagnosis because it is related to a wrong point of view, namely *P*(+∖1) or *P*(−∖0). Also SPIN and SNOUT should be avoided due to its conditional limitations related to prevalence and combination of SP and SE. PPV and NPV are related to the right point of view for clinical diagnosis, namely *P*(1∖+) or *P*(0∖−). When considering predictive values of diagnostic tests, one must however, recognise and accentuate the influence of the prevalence of the clinical event. Whereas the weakness of SE and SP is their relationships to the conditional statements P(+∖1) and P(−∖0),the problem with PPV and NPV is that they are predictive values largely dependent on the prior probability. Consequently, predictive values from one study should not be transferred to some other setting with different prevalences. Prevalence affects PPV and NPV differently. PPV is increasing, while NPV decreases with the increase of the prevalence. When the prevalence is very high, a negative test is most likely a false negative. When prevalence is very low, a positive test is most likely a false positive.

Predictive values have a clear meaning in clinical context. If a patient is tested positive with the O’Brien test for a SLAP lesion, the patient may ask: ‘Does this mean I have this clinical condition?’. ‘No’ you answer, ‘it is not certain for 100%. The patient ‘I understand that it is not totally clear, but what then is the chance that I have this problem?’ Given 26% as the highest prevalence presented in literature for a SLAP lesion and given a pooled estimate for SE of 0,66 and for SP 0,36, PPV is 0,27 and NPV is 0,75 (the contingency table is presented in Table 4).

**Table 4:** Contingency table for the O’Brien test with a SE of 0.66 and SP of 0.36 under prevalence of 0.26

Prevalence = 0.26		SLAP lesion			
O’Brien test		1	0		
+		d1 = 17	d0 = 47	d = 65	PPV = 0.27
–		h1 = 9	h0 = 27	h = 35	NPV = 0.75
		n1 = 26	n0 = 74	*n* = 100	
		SE = 0.66	SP = 0.36		

You answer the patient: ‘In 100 persons presenting positively on the test, 30 of them will have the clinical condition. What then is the ‘negative predictive value’? This is 0.8, whereby with a negative test result there is a 20% chance of presence of the clinical condition, which is also called the post-test probability of a negative test’ (1–0.8).

Because the PPV and NPV are dependent on the pre-test probability, these scores are termed post-test probabilities. At first sight, the PPV and NPV are measures which respond to the clinical relevant question: ‘what is the chance that the clinical condition will be present or absent in context of a positive or negative test result?’. However, the interpretation of the PPV and NPV is limited to populations with the same prevalence of clinical condition as the specific population to which the patient belongs. The prevalence in a clinical setting may differ considerably between for instance primary care practice and hospital. Patients in primary care practice will generally have the clinical condition at an earlier and milder stage.

Due to its influence and unknown differences between clinical settings, prevalence is the nemesis in the application of the predictive values. Therefore, another variable has been introduced to evaluate the strength of a diagnostic test, namely the likelihood ratio. Likelihood ratios determine how much more likely a particular test result is among people who have the clinical condition of interest than it is among people who do not have the condition. LIKELIHOOD RATIO (LR) is the ratio of two probabilities.

The positive likelihood ratio (LR+) is the ratio between the proportion of the individuals having the clinical status and presenting a positive test result to the proportion of the individuals not having the clinical condition but presenting with a positive test result: POSITIVE LIKELIHOOD RATIO = LR+ =$$ \frac{P\left(+\setminus 1\right)}{P\left(+\setminus 0\right)} $$ = $$ \frac{\raisebox{1ex}{$d1$}\!\left/ \!\raisebox{-1ex}{$n1$}\right.}{\raisebox{1ex}{$d0$}\!\left/ \!\raisebox{-1ex}{$n0$}\right.} $$ .

A positive likelihood ratio is a measure of how much more likely a positive test result is among people who have the condition of interest than it is among people who do not have the condition of interest. For instance, with the data presented in Table 4, LR+ = 1.03, meaning that the likelihood of a positive outcome of the O’Brien test in patients with a SLAP lesion is only 3% higher than in patients without SLAP lesions.

The negative likelihood ratio (LR-) is the ratio between the proportion of the individuals having the clinical status and presenting a negative result on the clinical test to the proportion of the individuals not having the clinical condition and presenting with a negative test result: $$ \mathrm{NEGATIVE}\ \mathrm{LIKELIHOOD}\ \mathrm{RATIO}=\mathrm{LR}-\frac{P\left(-\setminus 1\right)}{P\left(-\setminus 0\right)}=\frac{\raisebox{1ex}{$h1$}\!\left/ \!\raisebox{-1ex}{$n1$}\right.}{\raisebox{1ex}{$h0$}\!\left/ \!\raisebox{-1ex}{$n0$}\right.} $$.

A negative likelihood ratio is a measure of how much more likely a negative test result is among people who have the condition of interest than it is among people who do not have the condition of interest.

With $\mathrm{SE}=\frac{d1}{n1}$ and $\mathrm{SP}=\frac{h0}{n0}=\frac{n0-d0}{n0}$ it can be deduced that $\frac{d0}{n0}=1-\mathrm{SP}$ . This gives for $\mathrm{LR}+=\frac{SE}{1- SP}$ and analogously for $\mathrm{LR}-=\frac{1- SE}{SP}$ . LR+ is related to the concept ‘ruling IN the disease’, LR- to ‘ruling OUT the disease’. Likelihood ratios of 1 indicate that the test is uninformative.

A LR+ bigger than 1 means that the probability for presence of the clinical entity is more than chance (head or tail). A LR- smaller than 1 means that the probability of absence of the clinical entity is bigger than head or tail chance. LR+ ranges from 1 to infinity, LR- from 0 to 1. LRs have a strong power because LR+ and LR- are independent of the prevalence in the population. Likelihood ratios have a number of potencies. First, LRs can be combined with the pre-test probability to calculate the post-test predictive values PPV and NPV using formulas based on Bayes’s theorem [10]:

With $\uplambda =\frac{\frac{prevalence}{\left(1- prevalence\right)}\ast LR}{\frac{prevalence}{\left(1- prevalence\right)}\ast LR+1}$, PPV = λ (with LR = LR+) and NPV = 1- λ (with LR = LR-). Furthermore, LRs are applicable in populations in which the clinical condition may have a different prevalence to the population from which the likelihood ratio was calculated.

With a likelihood ratio nomogram post-test, probabilities can be deduced from the pre-test probability and the LR [10].

To avoid the calculations to find the shift from prior probability) to posterior probability (PVs), McGee (2002) described a simpler method (under the condition of prevalence between 10 and 90%) to interpret LRs using (so called ‘bedside’) estimates of approximate change in probability of the clinical event (in %) accurate to within 10% for a prevalence between 10 and 90% [11].

A LR+ bigger than 10, indicating an estimated shift in probability of at least 45%, has been stated to be strongly indicative for the presence of a clinical entity, between 5 and 10 moderate (estimated shift of at least 30%) and between 2 and 5 weak (estimated shift 15%) [10, 11]. A LR- less than 0.1 is strongly indicative for absence of the clinical entity (estimated shift at least 45%), between 0.1 and 0.2 moderate (estimated shift at least 30%) and between 0.2 and 0.5 weak (estimated shift at least 15%) [10]. However, in musculoskeletal disorders, the LRs hardly approach a maximum of 4 for LR^+^ and 0.25 for LR^−^. For instance, in their recent meta-analysis on physical examination tests of the shoulder, Gismervik et al. (2017) presented pooled results of LR^+^s ranging between 0.67 and 3.91 (with those being bigger than 1 between 1.03 and 3.91). LR^−^s ranged between 0.63 and 1.06 (with those smaller than 1 between 0.57 and 0.91) [4]. Table 5 presents the increase in probability (i.e. posterior probability – prior probability = posttest probability – prevalence) as related to a Monte Carlo representation of combinations between SE and SP. To accentuate is the decrease in probability under the condition of SE 0.4 and SP 0.4 for the LR+ as well as for the LR-.

**Table 5:** Probability increases (%) for P(1∖+) and for P(0∖-) in relation to prevalence and LRs

			PPV				probability increase (%) for P(1∖+)			
			prevalence				prevalence			
SE	SP	LR+	0.2	0.4	0.6	0.8	0.2	0.4	0.6	0.8
0.8	0.8	4.00	0.50	0.73	0.86	0.94	0.30	0.33	0.26	0.14
0.4	0.8	2.00	0.33	0.57	0.75	0.89	0.13	0.17	0.15	0.09
0.8	0.4	1.33	0.25	0.47	0.67	0.84	0.05	0.07	0.07	0.04
0.4	0.4	0.67	0.14	0.31	0.50	0.73	−0.36	−0.42	−0.36	−0.21
			NPV				probability increase (%) for P(0∖-)			
			prevalence = P [1]				1-prevalence = P(0)			
SE	SP	LR-	0.2	0.4	0.6	0.8	0.8	0.6	0.4	0.2
0.8	0.8	0.25	0.94	0.86	0.73	0.50	0.14	0.26	0.33	0.30
0.4	0.8	0.75	0.84	0.67	0.47	0.25	0.04	0.07	0.07	0.05
0.8	0.4	0.50	0.89	0.75	0.57	0.33	0.09	0.15	0.17	0.13
0.4	0.4	1.50	0.73	0.50	0.31	0.14	−0.07	−0.10	−0.09	−0.06

Diagnostic test calculators are available on the internet to determine SE, SP, LRs and or to determine post-test probabilities given prevalence and test characteristics [12, 13].

To return to our patient who tested positive on the O’Brien’s test. With a prevalence of 026, a SE of 0.66 and a SP of 0.36, the LR+ of the O’Brien test is only 1.03, i.e. very weak. The test increases the prior probability (prevalence) of 0.26 up to a posterior probability (PPV) of 0.27, an increase of only 1%, And with the prior probability P(0) of 0.8 reduced to a NPV of 0.75, this makes the O’Brien test worthless under the condition of the 0.26 prevalence. To be sure, you need to advice the patient to proceed with other investigations (medical imaging).

As stated above, the main nemesis in clinical diagnosis is the pre-test probability (prevalence). This must not be restricted to the prevalence of the clinical event in the population. Based on the anamnestic information, the physiotherapist may think of a particular clinical problem (primary hypothesis) in varying degrees of likeliness. Standard screening and red flags questions may have ruled out specific conditions. Once the primary hypothesis of clinical condition is expressed, a conscious levelling of the prior probability of this condition can be made.

### Take home messages

- Therapists should not rely on the SNOUT and SPIN mnemomics.
- Prevalence is the nemesis in diagnosis but can be ‘upgraded’ based on anamnesis and the levelling of a prior probability.
- PPV and NPV are dependent on SE, SP and prevalence.
- With λ = $\frac{\frac{prevalence}{\left(1- prevalence\right)}\ast LR}{\frac{prevalence}{\left(1- prevalence\right)}\ast LR+1}$, the best approach is to use the LRs in combination with a prevalence estimation to calculate the PPV (PPV=PV) and NPV (NPV = 1-PV) [10].

## Abbreviations

**∩**: Boolean operator ‘and‘
DA: Diagnostic accuracy
FNF: True Negative Fraction
FPF: False Positive Fraction
LR: Likelihood ratio
NPV: Negative Predictive Value
P: Probability
PPV: Positive predictive value
PV: Post test probability
SE: Sensitivity
SLAP: Superior labrum anterior and posterior
SNOUT: Sensitive test when Negative rules OUT the disease
SP: Specificity
SPIN: Specific test when Positive rules IN the disease
TNF: True negative fraction
TPF: True positive fraction
